# A Case of Chronic Enlargement of the Spleen, with Remarks

**Published:** 1840-08

**Authors:** Richard R. Dashiell

**Affiliations:** Tennessee


Art. IV.—*A Case of Chronic Enlargement of the Spleen, with Remarks.* By Richard R. Dashiell, M. D., of Tennessee.

The case which I am about to relate is one of not very unfrequent occurrence in this State. I allude to the chronic enlargement of the spleen, succeeding an attack of intermittent fever.

*Elizabeth Harris*, æt. 36, was subject to periodical attacks of ague and fever for fifteen years, and upon the cessation of this disease, about six years ago, she complained of pain in the left hypochondrium, which extended to her back; from which time the spleen increased in size until it has attained its present dimensions. She has been pregnant five times, and at the last accouchement gave birth to twins, from which time the pain in her left side increased. When I first saw her, she had that peculiar hue of skin which often follows tedious intermittents, and which those who have seen it will always recognise. This color is easily distinguished from the hue of slight jaundice—it is what is usually termed a clay-colour. She had cough, general debility, loss of appetite, pain in the head and back, periodical blindness, vertigo, constipation, loss of sleep, sometimes for a week at a time; her catamenial discharge was irregular, being frequently merely a show, and then entirely suppressed. She had no fever; pulse was rather slow and regular; her belly enormously tumefied. Upon first inspection, I accused her of being pregnant, which imputation she resented warmly, as she had been separated from her husband for six years. On a more minute manipulation, I found that the abdominal tumefaction did not depend upon the presence of a fœtus in utero, or of fluid in the peritoneum, but was produced by enlargement of the spleen, and was a case of what is vulgarly called 'Ague Cake.' The tumour was remarkable for its dimensions, occupying more than one-half of the abdominal cavity, and extending to the spine of the ilium. Judging from the impression it gave through the integuments, it must have been three or four inches thick. At one time she describes it as having been as large as "a clever sized watermelon."

As there was tenderness upon pressure, I applied three cups over the splenic region, and recommended a light diet, in addition to which, I directed a blue pill every other night. The pills were continued until the tenderness was removed, when I inserted two setons, which produced rapid diminution of the enlargement, and a perfect cure, so far as the spleen was concerned.—With respect to tonics, I found them of great service. I used a 'decoction of the woods,' with supertartrate of iron. They no doubt gave vigor to the constitution, which had been impaired by long ill health, and tended to promote the absorption of the morbid products, on which the elargement chiefly depends.

In conclusion, I would offer the suggestion, whether the reason why enlargements of this viscus from *sanguineous engorgement* are not more frequently cured, is, that they are confounded with enlargements arising from *inflammatory action*, which have resisted all the means of treatment devised for their cure by the 'masters of the healing art.' The latter, or enlargements arising from inflammatory action, are generally hard to the touch, and are always more or less painful—on manipulation they present the feeling of *induration*, though sometimes only in spots, particularly in the inferior and left lateral portions, the right being free from any appreciable lesion. On the contrary, enlargements arising from sanguineous engorgement, have a soft, spongy, elastic feeling, during the absence of febrile excitement, whilst during the progress of the fever they impress us with the sense of *distension*, and not of hardness.

*Chronic indurations* of the spleen have resisted every plan of treatment. Iodine and mercury used in every form, have been alike unsuccessful. Temporary relief only has been afforded by cupping or leeching, low diet, change of residence, mineral-water bathings, keeping the bowels free, and wearing the tartar emetic plaster over the left hypochondrium. On the other hand, *chronic enlargements* of the spleen are capable of reduction by adopting a hygienic course of living, by strengthening the system by the exhibition of tonics, by an alterative course of mercury, and finally, by the use of the seton. It is this condition of the spleen alone that yields to a judicious treatment, while all others remain the opprobria of our art.

*July*, 1840.

